# Phosphorylation Promotes the Accumulation of PERIOD Protein Foci

**DOI:** 10.34133/research.0139

**Published:** 2023-05-05

**Authors:** Mengna Li, Shujing Li, Luoying Zhang

**Affiliations:** ^1^ Key Laboratory of Molecular Biophysics of Ministry of Education, College of Life Science and Technology, Huazhong University of Science and Technology, Wuhan, Hubei 430074, China.; ^2^ Department of Life Sciences, Bengbu Medical College, Bengbu, Anhui 233030, China.; ^3^ Hubei Province Key Laboratory of Oral and Maxillofacial Development and Regeneration, Wuhan, Hubei 430022, China.

## Abstract

Circadian clock drives the 24-h rhythm in our behavior and physiology. The molecular clock consists of a series of transcriptional/translational feedback loops operated by a number of clock genes. A very recent study reported that the clock protein PERIOD (PER) is organized into discrete foci at the nuclear envelope in fly circadian neurons, which is believed to be important for controlling the subcellular localization of clock genes. Loss of inner nuclear membrane protein lamin B receptor (LBR) leads to disruption of these foci, but how they are regulated is yet unknown. Here, we found that PER foci are likely phase-separated condensates, the formation of which is mediated by intrinsically disordered region in PER. Phosphorylation promotes the accumulation of these foci. Protein phosphatase 2A, which is known to dephosphorylate PER, hampers the accumulation of the foci. On the other hand, the circadian kinase DOUBLETIME (DBT) which phosphorylates PER enhances the accumulation of the foci. LBR likely facilitates PER foci accumulation by destabilizing the catalytic subunit of protein phosphatase 2A, MICROTUBULE STAR (MTS). In conclusion, here, we demonstrate a key role for phosphorylation in promoting the accumulation of PER foci, while LBR modulates this process by impinging on the circadian phosphatase MTS.

## Introduction

Most if not all organisms on the earth display circadian rhythms, manifested in various aspects of physiology and behavior. These rhythms are driven by endogenous circadian clocks, which at the molecular level consist of a series of transcriptional/translational feedback loops operated by a number of core clock genes [1]. In fruit flies, these loops center on 2 transcription factors CLOCK (CLK) and CYCLE (CYC) that heterodimerize and activate the transcription of other core clock genes via E-box elements in the genome, including *period* (*per*) and *timeless* (*tim*). PER and TIM protein are synthesized in the cytoplasm, translocate into the nucleus, and repress the transcriptional activities of CLK/CYC, thus repressing their own transcription. PER and TIM also undergo a series of posttranslational modifications, most importantly phosphorylation, which ultimately lead to their degradation [2]. Once PER and TIM are degraded, the repressive influences on CLK/CYC are removed and they can initiate a new round of transcription. These 4 proteins constitute the major loop of *Drosophila* clock, and the speed at which this loop operates determines the period of circadian rhythm at behavior and physiological levels.

A previous study reported that PER accumulates in discrete foci in the cytoplasm of cultured fly Schneider 2 (S2) cells, but this observation did not draw much attention as it was uncertain whether these foci are overexpression artifacts [3]. Very recently, Xiao et al. [4] tagged the endogenous PER with fluorescent labels and discovered that PER forms discrete foci near the nuclear lamina in fly circadian neurons. These foci are circadian controlled and may play a role in positioning core clock genes near the nuclear periphery where their transcription is repressed. Loss of lamin B receptor (LBR), an inner nuclear membrane protein, leads to disruption of PER foci formation and the nuclear periphery localization of the *per* gene. However, almost nothing is known regarding how these foci form and accumulate in the cell. In addition, we and others have reported that LBR regulates circadian rhythm in flies and human cells, but the mechanism has not been elucidated [4,5].

Here, we found that PER foci are highly likely to be phase-separated condensates formed by interactions of intrinsic disordered region (IDR) in PER. Protein phosphatase 2A (PP2A), known to dephosphorylate and stabilize PER protein, hampers the accumulation of PER foci both in vivo and in S2 cells, whereas inhibiting PP2A facilitates PER foci accumulation [6]. Consistently, overexpressing DOUBLETIME (DBT), a circadian kinase known to phosphorylate PER, enhances PER foci accumulation [7]. LBR binds to the catalytic subunit of PP2A, MICROTUBULE STAR (MTS), and functions to destabilizes MTS specifically in the nucleus, thereby influencing nuclear PER foci accumulation. Taken together, these results unveil a mechanism centering on phosphorylation that facilitates PER foci accumulation, while LBR participates in this process by influencing the stability of MTS.

## Results

### PER IDR forms phase-separated condensates

We employed S2 cells to characterize the nature of PER foci and were able to observe them both by immunostaining and live imaging (Fig. S1A), consistent with the previous report [3]. To verify that these foci are similar to those in vivo and are not merely an artifact of overexpression, we compared the level of PER protein in S2 cells with that of wild-type fly heads collected at Circadian Time 0 (CT0, defined as the time of subjective lights on) on the first day of constant darkness (DD1), as it has been reported that the foci show most prominent accumulation at this time point [4]. We found that S2 cells transfected with 100 ng *per* complementary DNA (cDNA) display PER protein level comparable with that in vivo (Fig. S1B). Then, we treated cells with 1,6-hexanediol, which disrupts weak hydrophobic interactions, and found this leads to disassembly of the foci (Fig. S1C) [8]. These results indicate that the PER foci in S2 cells display liquid-like properties, similar to what has been reported for the foci in vivo [4].

**Fig. 1.**
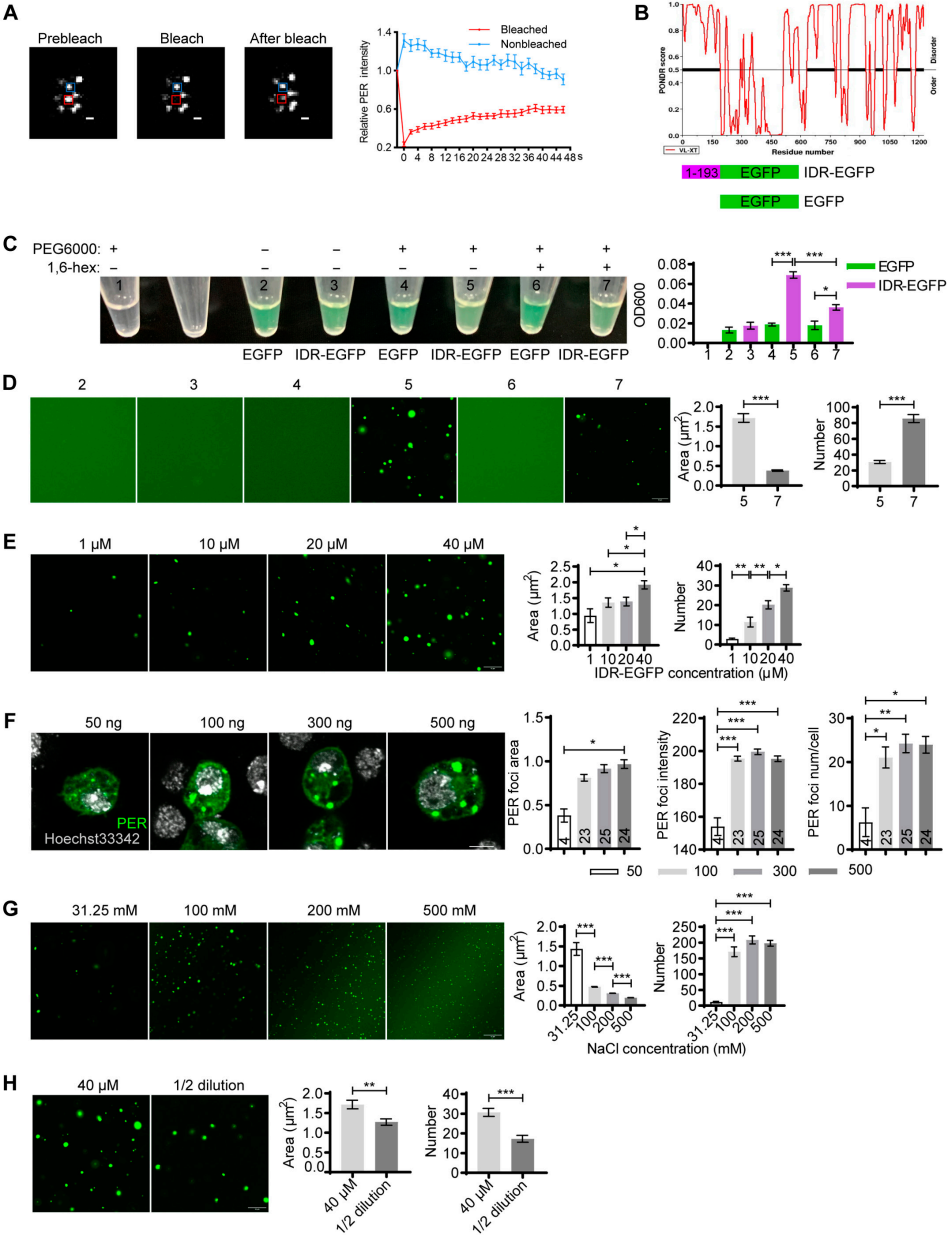
PER IDR forms phase-separated condensates. (A) Left panel: Representative confocal images of the PER foci FRAP assay in S2 cells transfected with pAc-*per*-EGFP-V5-HisB for 36 h. Red and blue rectangles represent bleached foci and control, respectively. Scale bar, 1 μm. Right panel: Quantifications of fluorescence intensity. *n* = 15 cells. (B) Top panel: Disordered region analysis of PER protein using PONDR. Black with bold lines indicate that disordered sequences. Bottom panel: Schematics of recombinant protein. (C) Left panel: Representative image demonstrating the turbidity of 40 μM solution of indicated contents with or without 10% PEG6000 and 10% 1,6-hexanediol (simplified as 1,6-hex). Each solution is given a number as an indicator. Right panel: Plot shows mean optical density 600 (OD600) of the solutions. *n* = 3. (D) Left panel: Representative confocal images of protein solutions in (B). Right panel: Quantification of the area and number of IDR-EGFP droplets in the presence PEG6000 or PEG6000 and 1,6-hexanediol. *n* ≥ 10 fields of view from 3 repeats. Scale bar, 10 μm. (E) Left panel: Representative confocal images of IDR-EGFP droplets at different protein concentrations. IDR-EGFP was diluted with buffer to indicated concentrations. Right panel: Quantification of the area and number of droplets. *n* ≥ 10 fields of view from 3 repeats. Scale bar, 10 μm. (F) Left panel: Representative confocal images of S2 cells transfected with indicated dosage of pAc-*per*-V5-HisB for 36 h and immunostained with Hoechst 33342 (gray) and anti-PER (green). Right panel: Quantification of PER foci area, intensity, and number per cell. *n* refers to the number of cells and is indicated on the bars. (G) Left panel: Representative confocal images of PER-IDR droplets at different NaCl concentrations. Protein concentration used is 10 μM. Right panel: Quantification of the area and number of droplets. *n* ≥ 10 fields of view from 3 repeats. Scale bar, 10 μm. (H) Left panel: Representative confocal images of 40 μM PER-IDR droplets and 1/2 dilution. Right panel: Quantification of the area and number of droplets. *n* ≥ 10 fields of view from 3 repeats. Scale bar, 10 μm. Error bars represent standard error of the mean (SEM). One-way ANOVA, Tukey’s multiple comparison test for (C) and (E) to (G). Student *t* test for (D) and (H). *P < 0.05, **P < 0.01, ***P < 0.001.

We further investigated the liquid-like properties of PER foci by measuring the rate of fluorescence recovery after photobleaching (FRAP). We performed FRAP experiments on PER-EGFP foci in S2 cells. After photobleaching, fluorescence partially recovered on scale of seconds (Fig. 1A and Movie S1), which indicates that they are dynamic. Since PER bears multiple IDRs as shown in past studies and IDRs can facilitate condensate formation, we tested whether these PER foci are phase-separated condensates [4,8,9]. We selected the longest IDR (1 to 193), fused it to EGFP and expressed and purified it in a bacterial system (Fig. 1B and Fig. S2A). Enhanced green fluorescent protein (EGFP) and IDR-EGFP were added to 10% polyethelene glycol-6000 (PEG6000) solution, which increases macromolecular crowding and promotes phase separation. IDR-EGFP rendered the solution opaque, while EGFP solution remained clear (Fig. 1C). We examined the solutions and observed EGFP-positive micron-sized droplets (Fig. 1D). Live imaging demonstrates that the droplets are highly dynamic and 2 droplets can fuse into 1 (Movie S2).

Phase-separated droplets typically scale up in size with increased concentration [10]. Here, we performed droplet formation assay with varying concentrations of IDR-EGFP ranging from 1 to 40 μM. Indeed, both the size and number of the droplets increase at higher concentrations (Fig. 1E). Similarly, the size, intensity, and number of PER foci in S2 cells also increase in a dose-dependent manner (Fig. 1F).

To investigate the biochemical properties of these IDR-EGFP droplets, we first treated them with 1,6-hexanediol. This substantially reduced the opacity of IDR-EGFP solution and the droplets size, as well as increased the number of the droplets (Fig. 1C and D). In addition, we found that 2 h after the formation of the droplets, they start to exhibit fibrous structure but are still sensitive to 1,6-hexanediol (Fig. S2B). We next assessed the ability of IDR-EGFP to form droplets under varying salt concentrations that perturb electrostatic interactions. The size of the droplets decreases as salt concentration increases from 31.25 to 500 mM (Fig. 1G). These observations implicate that both hydrophobic and electrostatic interactions contribute to the formation of PER IDR condensates.

Lastly, we tested whether the IDR-EGFP droplets are reversible. We first allowed the droplets to form, and then the protein concentration was diluted by half. The size and number of the droplets exhibited substantial reduction (Fig. 1H). These findings demonstrate that PER IDR can form droplets with a distribution of sizes depending on the condition of the system. Once formed, these droplets respond to changes in the system by rapidly altering their size and number. These features are characteristic of phase-separated condensates formed by weak protein–protein interactions [8].

These findings strongly suggest the sufficiency of IDR (1 to 193) in triggering the formation of phase-separated droplets. Next, we investigated the necessity of this fragment by generating PER without these N-terminal sequences (194 to 1,224). Foci can still be observed in S2 cells expressing this truncated version of PER, which are sensitive to 1,6-hexanediol as well (Fig. S2C). This indicates that IDR 1 to 193 is not required for the formation of phase-separated droplets. We reason that this may be because PER contains multiple IDRs and thus loss of IDR (1 to 193) does not eliminate the ability of PER protein to form phase-separated condensates (Fig. S2D).

In summary, these series of results demonstrate that PER IDR can form phase-separated condensates in PEG solution, which may contribute to the formation of PER foci in vivo.

### PP2A reduces PER foci accumulation

**Fig. 2.**
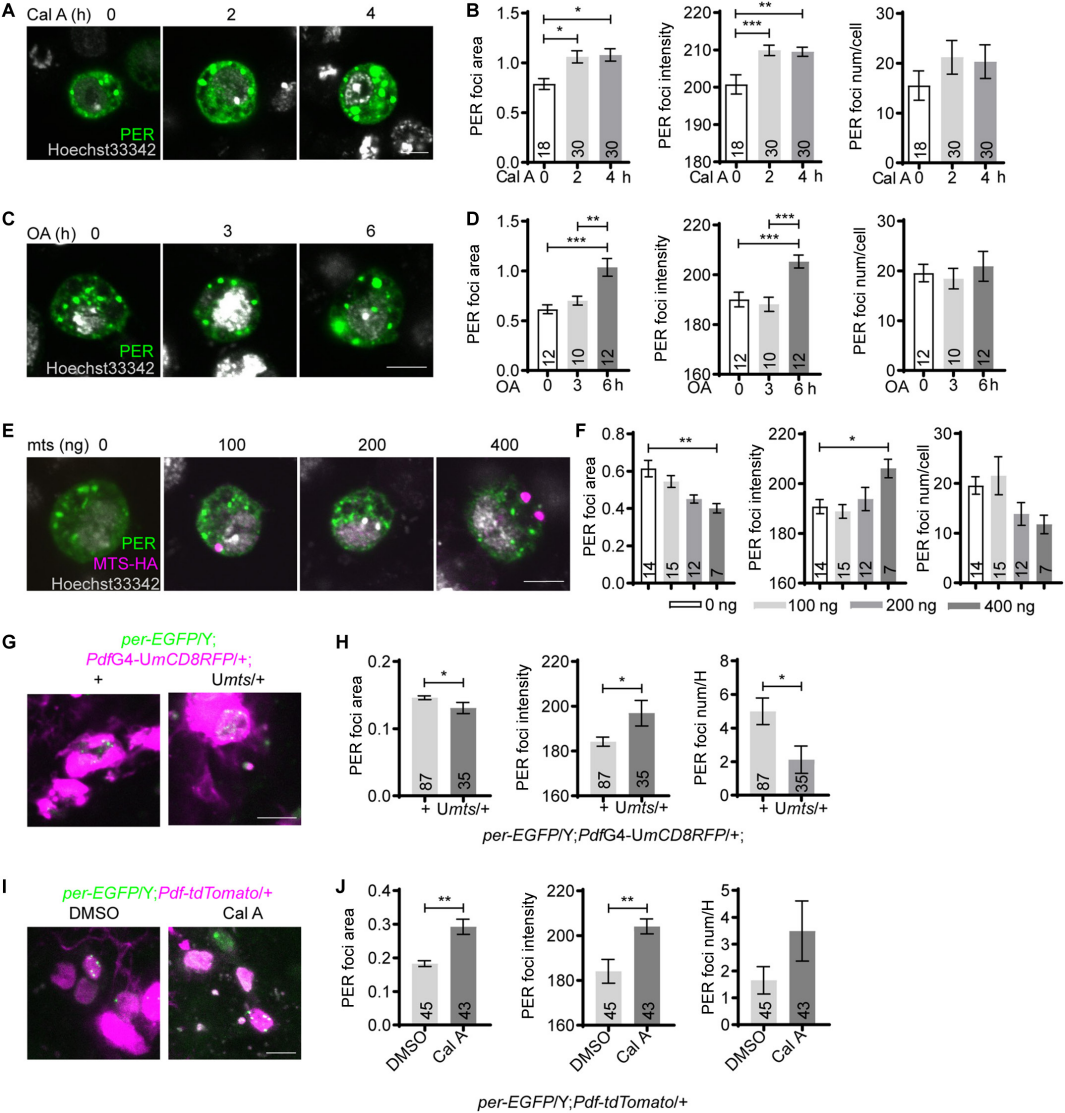
MTS reduces PER foci accumulation. (A) Representative confocal images of PER foci in S2 cells transfected with 100 ng of pAc-*per*-V5-HisB for 30 h and then treated with Cal A (30 nM) for indicated time periods. The cells were subsequently immunostained with Hoechst 33342 (gray) and PER antibody (green). (B) Quantification of PER foci area, intensity, and number per cell in (A). (C) Representative confocal images of PER foci in S2 cells transfected with 100 ng of pAc-*per*-V5-HisB for 30 h and treated with okadaic acid (OA, 5 nM) for indicated time periods. The cells were then immunostained with Hoechst 33342 (gray) and PER antibody (green). (D) Quantification of PER foci area, intensity, and number per cell in (C). (E) Representative confocal images of PER foci in S2 cells cotransfected with 100 ng pAc-*per*-V5-HisB and indicated dosage of pMT-*mts*-HA for 36 h. The cells were then immune-stained with Hoechst 33342 (gray), PER antibody (green) and HA antibody (magenta). (F) Quantification of the PER foci area, intensity, and number per cell in (E). (G) Representative confocal images of PER foci in the s-LNvs of *per*-EGFP/Y;*Pdf*GAL4*-*UASmCD8RFP*/+* flies overexpressing *mts* and controls dissected at CT0 on DD1. Green, PER-EGFP; magenta, mCD8RFP. (H) Quantification of PER foci area, intensity, and number per hemisphere (num/H) in (G). (I) Representative confocal images of PER foci in the s-LNvs of *per*-EGFP/Y;*Pdf-tdTomato*/+ flies dissected at CT0 on DD1. The brains were incubated in PBS solution containing dimethyl sulfoxide (DMSO) or 30 nM Cal A for 1 h. Green, PER-EGFP; magenta, tdTOMATO. (J) Quantification of PER foci area, intensity, and number per hemisphere in (I). Scale bar, 5 μm. *n* number is indicated on the bars. For (B), (D), and (F), *n* refers to the number of cells. For (H) and (J), *n* refers to the number of hemispheres. Error bars represent SEM. Student *t* test was used in (H) and (J). One-way ANOVA and Tukey’s multiple comparison test were used in (B), (D), and (F). *P < 0.05, **P < 0.01, ***P < 0.001. G, GAL. U, UAS.

Phosphorylation plays key roles in regulating PER stability and function [2]. Given that phosphorylation can also regulate phase separation, we tested whether phosphorylation participates in modulating these foci [11]. We first treated S2 cells with phosphatase inhibitor calyculin A (Cal A), which has been shown to be able to increase the phosphorylation of PER [6]. This remarkably increases the size and intensity of the foci, with a tendency of increase for foci number (Fig. 2A and B). Since PP2A is believed to be a major phosphatase acting on PER, we treated cells with okadaic acid that inhibits PP2A with high affinity [6,12]. This also leads to enhanced foci size and intensity (Fig. 2C and D). In contrast, overexpressing *mts* significantly reduces the size of the foci in a dose-dependent manner, while foci number exhibits a trend of reduction as well (Fig. 2E and F).

Next, we validated these findings in vivo and focused on PER foci by monitoring PER-EGFP in the small ventral lateral neurons (s-LNvs), which are considered to be the master pacemaker neurons [13–16]. We overexpressed *mts* in these cells using a *pigment dispersing factor* (*Pdf*)GAL4 and assessed the size, intensity, and number of PER foci at CT0 [13,17]. We found that overexpressing *mts* significantly reduced the size and number of the foci (Fig. 2G and H). We also tried to inhibit MTS function by expressing a dominant-negative form of *mts* (dn*mts*), but in these flies, PER-EGFP signal is barely detectable due to reduction on PER stability as previously reported (Fig. S3) [6,17]. To resolve this issue, we treated fly brains with Cal A and found this substantially increased the size of PER foci with a trend of increase in foci number (Fig. 2 I and J), opposite to that of *mts* overexpression.

Taken together, these findings indicate that PP2A functions to impede the accumulation of PER foci.

### DBT enhances PER foci accumulation

**Fig. 3.**
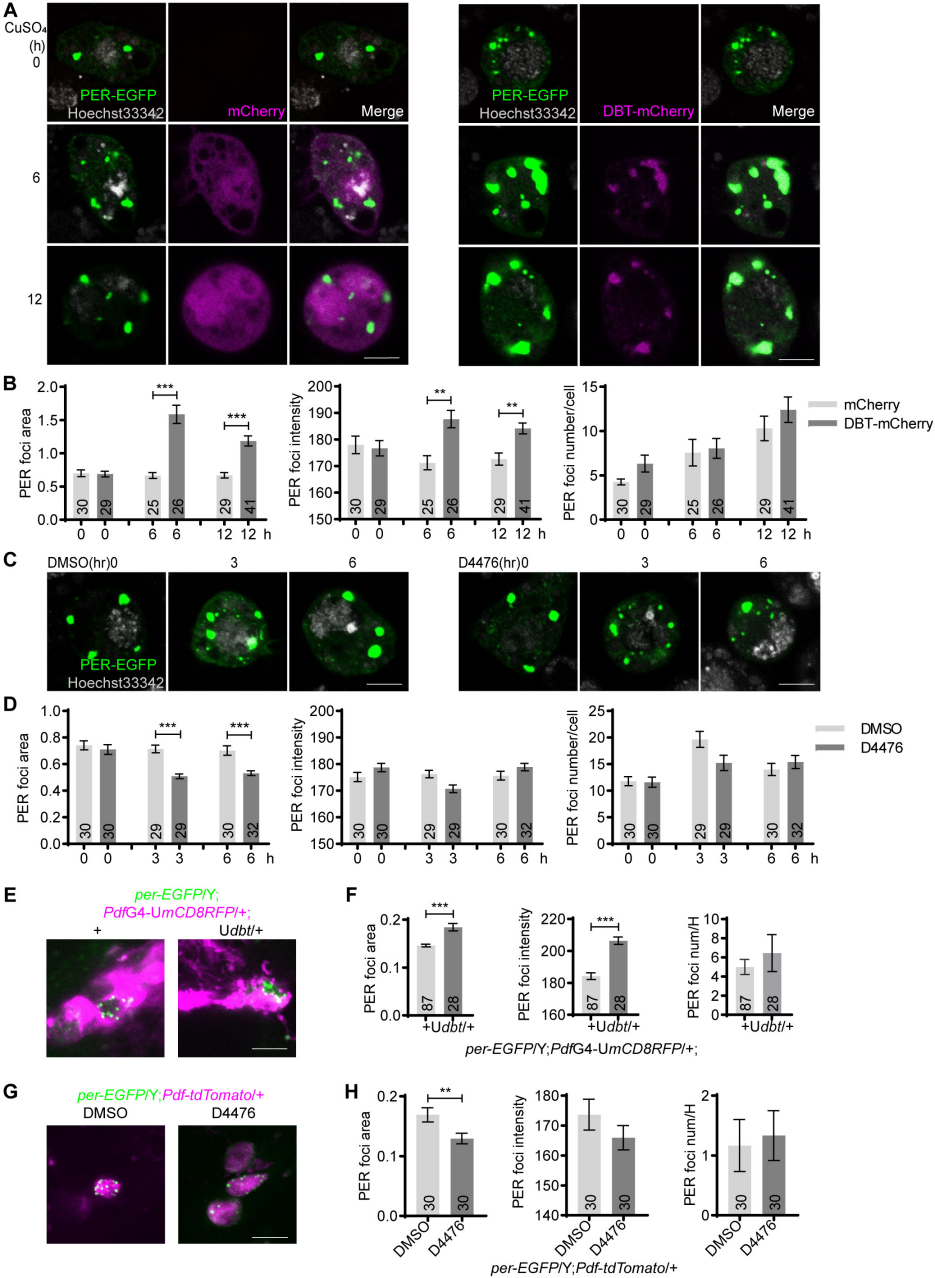
DBT increases PER foci size. (A) Representative confocal images of PER foci in S2 cells transfected with 100 ng of pAc-*per*-EGFP-V5-HisB and pMT-mCherry or pMT-*dbt-*mCherry for 30 h. Then, 500 μM CuSO_4_ was added to induce mCherry or *dbt-*mCherry expression. PER foci were live imaged at indicated time post induction. (B) Quantification of PER foci area, intensity, and number per cell in (A). (C) Representative confocal images of PER foci in S2 cells transfected with 100 ng pAc-*per*-EGFP-V5-HisB for 36 h and then treated with D4476 (10 μM) for indicated time periods. PER foci were imaged live at indicated time points. (D) Quantification of PER foci area, intensity, and number per cell in (C). (E) Representative confocal images of PER foci in the s-LNvs of *per-*EGFP/Y;*Pdf*GAL4*-*UASmCD8RFP*/+* flies overexpressing *dbt* and control flies at CT0 on DD1. Green, PER-EGFP; magenta, mCD8RFP. (F) Quantification of PER foci area, intensity, and number per hemisphere in (E). (G) Representative confocal images of PER foci in the s-LNvs of *per*-EGFP/Y;*Pdf-*tdTomato/+ flies dissected at CT0 on DD1. The brains were incubated in PBS solution containing DMSO or 20 μM D4476 for 1 h. Green, PER-EGFP; magenta, tdTOMATO. (H) Quantification of PER foci area, intensity, and number per hemisphere in (G). Scale bar, 5 μm. *n* refers to the number of cells (B and D) or hemispheres (F and H) and is indicated on the bars. Error bars represent SEM. One-way ANOVA and Tukey’s multiple comparison test was used for (B) and (D). Student *t* test was used for (F) and (H). *P < 0.01, **P < 0.01, ***P < 0.001.

The influences of PP2A on PER foci implicate that phosphorylation promotes the accumulation of these foci; therefore, we tested whether DBT, the major kinase that phosphorylates PER, modulates PER foci accumulation [7]. Overexpressing *dbt* in S2 cells results in obvious enhancement of foci size and intensity (Fig. 3A and B). Interestingly, we also observed a striking pattern of PER and DBT colocalization in the foci. Since DBT is the *Drosophila* homolog of mammalian casein kinase 1, we next treated the cells with a selective casein kinase 1 inhibitor, D4476 [18]. This substantially reduces PER foci size (Fig. 3C and D). Consistently, overexpressing *dbt* in the s-LNvs substantially enlarges the size of PER foci and increases the intensity, while D4476 treatment decreases foci size (Fig. 3E to H).

### LBR binds to and destabilizes MTS

Previous study identified LBR to be involved in foci formation/accumulation while knocking down *lbr* in all clock cells using a *tim*GAL4 driver substantially impairs locomotor rhythm [4]. Here, we further validated this by knocking down *lbr* using *Pdf*GAL4, *tim*GAL4, and a *cryptochrome* (*cry*)GAL4-16 that mainly drives expression in circadian neurons and assessed the effects on locomotor rhythm under constant darkness [19–21]. Knocking down *lbr* with *tim*GAL4 leads to the most prominent phenotype, including significantly lengthened period and reduced power value that is indicative of dampened rhythm (Table S1). At the cellular level, *lbr* deficiency significantly decreases the size and intensity of PER foci (Fig. S4A and B). We also attempted to investigate the effects of overexpressing *lbr*, but overexpression using *tim*GAL4 results in lethality and we failed to obtain adult flies (Table S1). Overexpressing *lbr* with *cry*GAL4-16 leads to severe morphological defects with severe loss of s-LNvs in the adult brain (Fig. S4C and D). Overexpression using *Pdf*GAL4, on the other hand, does not substantially alter locomotor rhythm (Table S1).

**Fig. 4.**
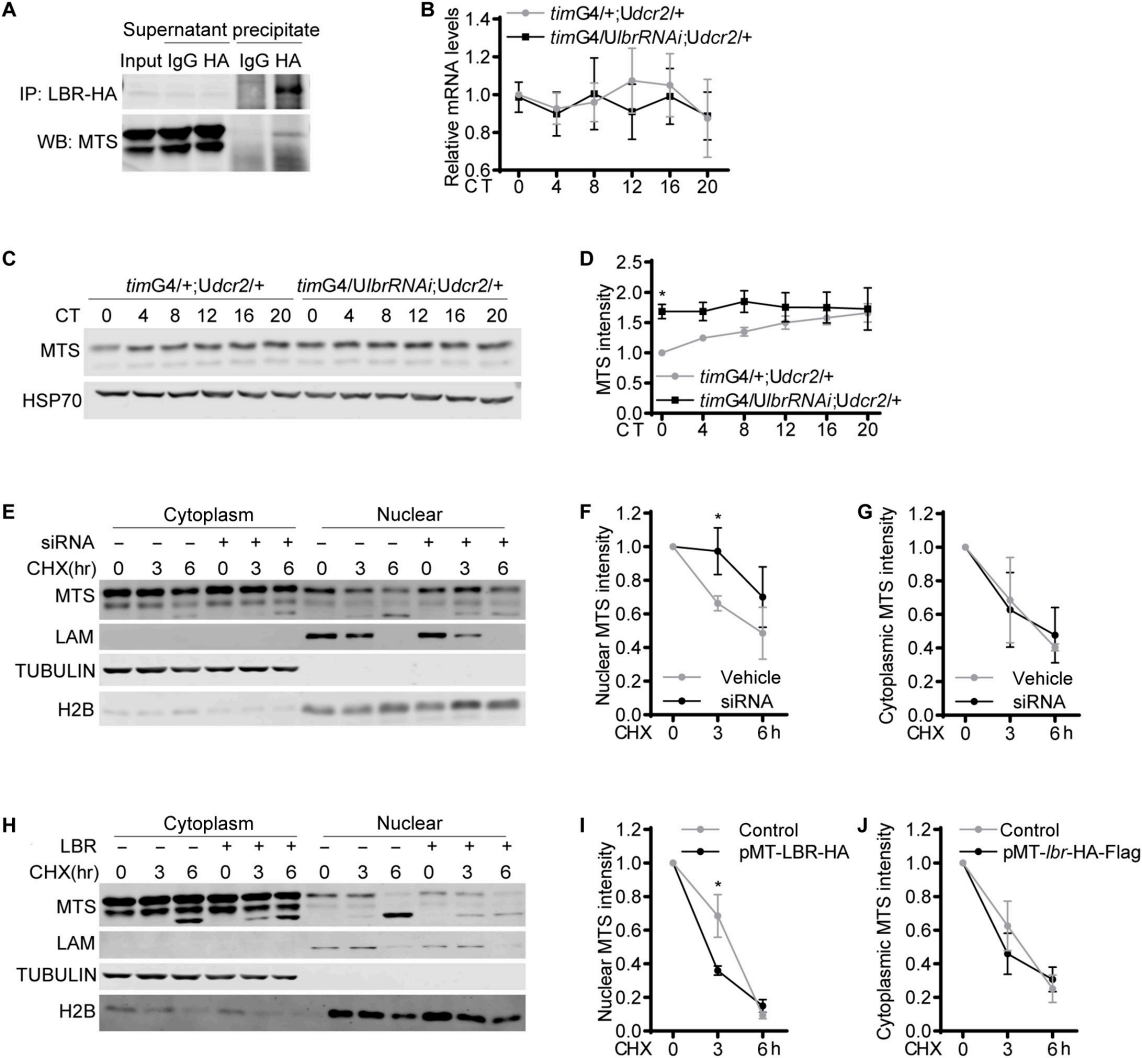
LBR binds to and destabilizes MTS. (A) Representative Western blots (WB) of protein extracts prepared from S2 cells transfected with pMT-*lbr*-Flag-HA and immunoprecipitates as well as supernatants. LBR was immunoprecipitated (IP) with HA antibody, and rabbit IgG was used as control. MTS was detected by Western blotting using MTS antibody. (B) Plot of relative mRNA abundance determined by qRT-PCR for *mts* from whole-head extracts of *tim*GAL4/+;UAS*dcr2*/+ and *tim*GAL4/UAS*lbrRNAi*;UAS*dcr2*/+ flies collected on the first day of constant darkness (DD1). *dcr2* was coexpressed to enhance the efficiency of RNAi. For each time series, the value of control group at CT0 was set to 1. (C) Representative Western blots of protein extracts prepared from whole heads of *tim*G4/+; U*dcr2*/+ and *tim*G4/UAS*lbrRNAi*;UAS*dcr2*/+ flies collected on DD1. HSP70 was used as a loading control. (D) Quantification of MTS level in (C), which was normalized to that of HSP70. For each group, the value of the control group at CT0 was set to 1. (E) Representative Western blots of cytoplasmic and nuclear extracts prepared from S2 cells transfected with *lbr* siRNA or control cells. The cells were treated with cycloheximide (CHX, 10 μg/ml) and harvested at the indicated time points. LAM, lamin. (F and G) Quantification of MTS level in nuclear (F) and cytoplasmic (G) fraction in (E). (H) Representative Western blots of cytoplasmic and nuclear extracts prepared from S2 cells transfected with pMT-*lbr*-Flag-HA or control cells. The cells were treated with cycloheximide (10 μg/ml) and harvested at the indicated time points. (I and J) Quantification of MTS level in nuclear (F) and cytoplasmic (G) fraction in (H). *n* = 3. Error bars represent SEM. Two-way ANOVA, Sidak’s multiple comparison test was used for (B) and (D). Student *t* test was used for (F), (G), (I), and (J). *P < 0.05. G, GAL; U, UAS.

To investigate the underlying mechanism by which LBR regulates PER foci accumulation, we first tested for direct physical interaction between LBR and PER. We coexpressed hemagglutinin (HA)-tagged LBR and PER in S2 cells and performed immunoprecipitation using HA antibody but were not able to detect PER in the precipitates (Fig. S5A). By searching the literature, we found that PP2A interacts with various components of the inner nuclear membrane including lamin, the binding partner of LBR [22–24]. We also observed that lamin coprecipitates with LBR while coprecipitation between lamin and MTS is barely detectable (Fig. S5B and C). Nonetheless, based on previous studies, we suspected that instead of directly regulating PER, LBR may exert effects on PER via PP2A. Consistent with this hypothesis, we observed in S2 cells that LBR coprecipitates with MTS (Fig. 4A). In flies with *lbr* knocked down in all clock cells using a *timeless*(*tim*)GAL4 driver [19], MTS protein level is significantly increased at the beginning of the day (Fig. 4B to D). This elevation in protein level appears to be caused by alteration at the posttranscriptional level, as the mRNA level of *mts* is not significantly altered. We further probed the mechanism by which LBR regulates MTS by treating S2 cells with cycloheximide, which inhibits protein synthesis, so we can observe the influences of LBR on MTS stability. We found that knocking down *lbr* delays MTS degradation in the nucleus but not the cytoplasm (Fig. 4E to G). On the other hand, overexpressing *lbr* hastens MTS degradation in the nucleus but not the cytoplasm (Fig. 4H to J). In addition, we noticed that knocking down *lbr* tends to speed up lamin degradation while overexpressing *lbr* significantly delays lamin degradation (Fig. S5D and E). Taken together, this series of results indicate that LBR acts to destabilize MTS in the nucleus, and this effect appears to be most prominent early in the day.

## Discussion

The observation that PER forms foci-like structure was first reported in the cytoplasm of S2 cells when PER is ectopically expressed [3]. In vivo, Xiao et al. [4] also found that PER foci first appears in the cytoplasm and then starts to accumulate in the nucleus. Our work here strongly suggests that PER foci are phase-separated condensates, and weak interactions of the IDRs of PER promote their formation. These condensates are initially of liquid-like state, but after a while, they appear to transition (at least partially) into a “glassy solid” state that are fibrous instead of round (Fig. S2B). Based on the literature, when condensates are in this state, they are more arrested but still reversible [8]. This less dynamic state may also account for the partial fluorescence recovery after photobleaching the PER foci in S2 cells.

Multiple studies have shown that phosphorylation can regulate phase separation both positively and negatively [11]. Here, we found that although phosphorylation does not appear to be required for PER IDR to form phase-separated condensates in vitro, it likely enhances PER foci formation, especially by increasing the size of PER foci. In S2 cells, we observed near-perfect colocalization of PER foci and DBT but not MTS, suggesting that DBT may coexist with PER in phase-separated condensates. Since phosphorylation of PER by DBT is known to play critical roles in determining PER protein stability, we suspect that one function of PER foci may be to concentrate PER for more efficient phosphorylation and stability regulation by DBT [2,7]. Obviously, much more needs to be done to characterize the contents and function of these foci. An ideal experiment would be to precisely disrupt PER foci with minimal effects on other aspects of PER function and other signaling pathways and then examine the effects on circadian rhythm. Unfortunately, thus far, we have not found a way to conduct such precise manipulations.

Notably, both inhibiting and overexpressing MTS result in increased PER foci intensity in S2 cells and in vivo, which seems to be a caveat. Our findings support the idea that inhibiting MTS enhances PER foci accumulation, thus contributing to an increase of PER foci size and intensity, accompanied by a trend of increase for foci number. On the other hand, MTS is known to stabilize PER protein, which may account for the enhanced PER foci intensity as overexpressing *mts* increases total PER protein level, but foci size and number are reduced [6]. This series of observations suggest that the direct influences of phosphorylation on PER foci accumulation is to modulate foci dimension and number.

LBR is one of the most important proteins in the inner nuclear membrane and is known to play critical roles in tethering heterochromatin to nuclear periphery during development and in cancer cells [24–26]. Xiao et al. reported that knocking down *lbr* in postmitotic neurons leads to dampening of fly locomotor rhythm, likely due to disturbance of PER foci accumulation [4]. Here, we validated their findings and further found that LBR targets MTS and facilitates its degradation most prominently at CT0, with a similar trend at CT4 and CT8 (Fig. 4C and D). This corresponds nicely with the peak accumulation of PER foci at CT0 followed by their gradual disappearance [4]. Consistently, MTS impedes PER foci accumulation both in vivo and in culture cells, which is opposite of the effects of LBR.

To our knowledge, LBR has not previously been shown to participate in modulating protein stability. We have reported that knocking down *lbr* reduces the protein level of lamin B1 (LMNB1, human homolog of *Drosophila* lamin) in human U2OS cells, while Gaudy-Marqueste et al. [27] demonstrated a reduction of LMNB1 level in human fibroblasts carrying *lbr* mutation. However, it is not yet clear whether the decrease of LMNB1 level associated with LBR deficiency is caused by enhanced degradation. Here, we observed that overexpressing *lbr* significantly delays lamin degradation, in line with previous findings in human cells. Protein degradation systems have been best described in the cytoplasm and the endoplasmic reticulum, but more recently, the nucleus has also emerged as a key compartment for ubiquitination and proteasome-mediated degradation [28,29]. Both the nucleus and inner nuclear membrane contain ubiquitination machinery, which could potentially mediate the effects of LBR on MTS [30]. Further molecular analysis will be required to delineate the detailed mechanism regarding how LBR regulates MTS stability.

**Fig. 5.**
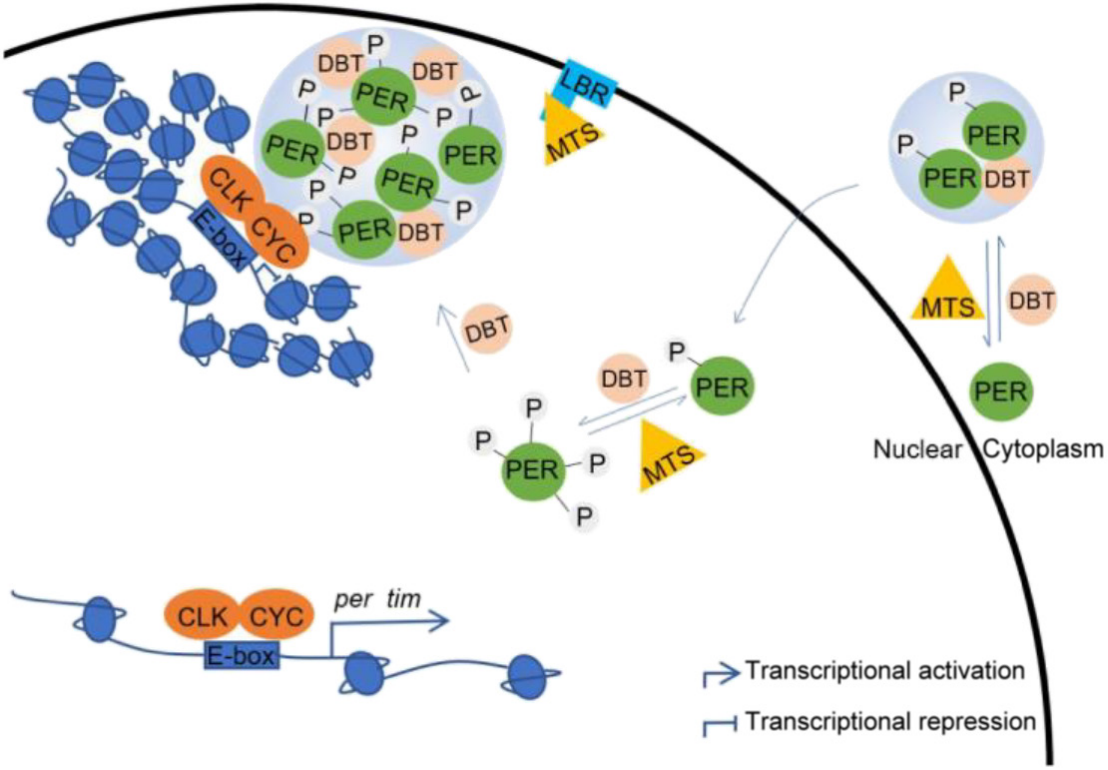
Model demonstrating the regulatory mechanism of PER foci. DBT phosphorylates PER and promotes PER foci accumulation, while MTS dephosphorylates PER and impedes PER foci accumulation. In the nucleus, PER foci localizes clock genes to the nuclear periphery where transcription is repressed. LBR binds to MTS and influences PER foci accumulation via MTS. P indicates phosphorylation.

In conclusion, we found PER IDR can form phase-separated condensates, which likely contributes to the development of PER foci in vivo. Phosphorylation appears to facilitate PER foci accumulation, with DBT increasing their size while MTS reducing their size. LBR promotes PER foci accumulation by destabilizing MTS, adding an extra layer of regulation on PER foci in the nucleus (Fig. 5).

## Materials and Methods

### Plasmids

The following plasmids are used in this study: pAc-*per*-V5-HisB [31], pMT-*lbr*-Flag-HA (FMO06243, *Drosophila* Genomics Resource Center), pMT-*mts*-Flag-HA (FMO02385, *Drosophila* Genomics Resource Center), pAc5.1/V5-HisB (V411020, Thermo Fisher Scientific), pAc-*per*-EGFP-V5-HisB, pAc-*per* (194-1224)-EGFP-V5-HisB, pMT-mCherry-Flag-His and pMT-*dbt*-mCherry-Flag-His. The open reading frame (ORF) of EGFP was amplified from pEGFP-C2 (#6083-1, Addgene) using upstream and downstream primers located at the BstBI and XbaI cleavage sites, respectively. EGFP ORF was then subcloned into pAc-*per*-V5-HisB such that EGFP is located at the carboxyl terminal end of *per*. EGFP ORF was also subcloned into pAc5.1/V5-HisB with the restriction enzymes XbaI and BstBI to acquire pAc5.1-EGFP-V5-HisB. The truncated PER (194 to 1,224 amino acids) with the ATG initiation codon was amplified from pAc-*per*-V5-HisB using upstream and downstream primers linked to the EcoRI and XbaI cleavage sites, respectively. The fragment was then subcloned into pAc5.1-EGFP-V5-HisB to acquire pAc-*per* (194-1224)-EGFP-V5-HisB. To generate pMT-mCherry-Flag-His, *dbt* with the stop codon was cleaved from pMT-*dbt*-Flag-His (a kind gift from Dr. Joanna Chiu at University of California, Davis) by EcoRI and XbaI, and then mCherry amplified from pCS2+8CmCherry (#34935, Addgene) by polymerase chain reaction (PCR) was ligated into the plasmid to obtain pMT-mCherry-Flag-His. The ORF of *dbt* was amplified from pMT-*dbt*-Flag-His and subcloned into pMT-mCherry-Flag-His with the *dbt* ORF at the amino terminal of the mCherry ORF.

### Fly culture

Flies were raised on standard cornmeal food at 25 °C and ~50% humidity under 12-h light/12-h dark (LD) cycles. The following fly strains were used in this study: *tim*GAL4 [19], UAS*dcr2* (V6009, Vienna *Drosophila* Resource Center), *w^1118^* (3605, Bloomington *Drosophila* Stock Center), UAS*lbrRNAi* (V110508, Vienna *Drosophila* Resource Center), UAS*lbr* (*lbr*^GS2162^, Kyoto Stock Center), *Pdf*GAL4 (6899, Bloomington *Drosophila* Stock Center), *cry*GAL4-16 (24514, Bloomington *Drosophila* Stock Center), *per*-AID-EGFP [16], UAS*mts* [17], UASdn*mts* [17], and UAS*dbt* [7]. All fly crosses were carried out at 25 °C, and male and female offspring were entrained at 25 °C for 3 d of LD followed by 1 d of constant darkness (DD) and collected randomly at the indicated time points. For *per*-AID-EGFP experiments, only male flies were used.

### Cell culture and transient transfection

*Drosophila* S2 cell line was cultured in Schneider’s medium (Gibco) supplemented with 10% fetal bovine serum and 1% penicillin–streptomycin (Thermo Fisher Scientific). The cells were plated in a 24-well plate and incubated for 24 h. Small interfering RNA (LBR: CGAAGACAATCTCAAATCT) and negative control were transiently transfected using riboFECTTM CP Regent (RIBOBIO C10511-05) according to the protocol provided by the manufacturer. Plasmids were transiently transfected using Lipofectamine 3000 Transfection Reagent (Thermo Fisher Scientific) according to the protocol provided by the manufacturer. Expression of *lbr*, *mts*, and *dbt* under pMT promoter was induced by adding 500 μM CuSO_4_ to the culture upon the completion of transfection and incubation for another 48 h unless specified otherwise. For experiments in which cycloheximide [10 mg/ml, MedChemExpress (MCE)], Cal A (30 nM, MCE), okadaic acid, sodium salt (5 nM, Sigma-Aldrich), and D4476 (10 or 20 μM, MCE) were used, they were added at the indicated time post transfection.

### RNA extraction and quantitative real-time PCR

Flies were entrained in LD for 3 d and collected on DD1 at the indicated time points and frozen immediately on dry ice. RNA extraction and quantitative real-time PCR (qRT-PCR) were conducted following our previously published methods [32]. In brief, total RNA was extracted from fly heads and S2 cells and then subjected to reverse transcription using TransScript One-Step gDNA Removal and cDNA Synthesis SuperMIX (TransGen Biotechnology). All qRT-PCRs were carried out on a Step One Plus Real-Time PCR System (Applied Biosystems, Life Technologies). Primers used are as follows: *rpl32*-f: 5′-TACAGGCCCAAGATCGTGAA-3′, *rpl32*-r: 5′-GCACTCTGTTGTCGATACCC-3′; *mts*-f: 5′-GCGACAAGGCCAAGGAGA3′, *mts*-r: 5′-AGTCGCCCATGAACAGGT-3′. *rpl32* is used as internal control.

### Protein extraction and Western blot

Flies were entrained in LD for 3 d and collected on DD1 at the indicated time points and frozen immediately on dry ice. Fly heads were separated by vortexing, and protein extracts were prepared by homogenizing using EB1 (20 mM Hepes [pH 7.5], 100 mM KCl, 5% glycerol, 2.5 mM EDTA, 5 mM dithiothreitol [DTT], 0.1% Triton X-100, and 25 mM NaF) supplemented with complete EDTA-free protease and phosphatase inhibitor cocktail (MCE). S2 cells were harvested and washed once with 1× phosphate-buffered saline (PBS) and then lysed with EB2 solution (20 mM Hepes [pH 7.5], 100 mM KCl, 5% glycerol, 5 mM EDTA, 1 mM DTT, 0.1% Triton X-100, and 25 mM NaF) supplemented with complete EDTA-free protease and phosphatase inhibitor cocktail (MCE). For cytoplasmic and nuclear extraction, the cytoplasmic fraction was obtained by CER1 (10 mM Hepes [pH 7.5], 10 mM KCl, 1.5 mM MgCl_2_, 0.34 M sucrose, and 10% glycerol) supplemented with complete EDTA-free protease and phosphatase inhibitor cocktail (MCE). S2 cells were vortexed vigorously for 15 s and incubated on ice for 10 min. NP-40 (3%) was added to the homogenates and vortexed for 5 s. This was followed by incubation on ice for 1 min and centrifugation at 16,000 g for 5 min at 4 °C. The supernatant that is the cytoplasmic fraction was transferred to a new prechilled tube. The pellet was suspended with EB2 solution supplemented with complete EDTA-free protease and phosphatase inhibitor cocktail (MCE). The homogenate was vortexed for 15 s every 10 min for a total of 40 min and centrifuged at 16,000 g for 5 min at 4 °C. The supernatant acquired is the nuclear fraction.

Equal amounts of protein were run on 10% sodium dodecyl sulfate–polyacrylamide gel electrophoresis gels and then transferred to nitrocellulose membranes. After incubation with primary antibodies at 4 °C overnight, membranes were incubated with secondary antibodies at room temperature for 1 h. The primary antibodies used were mouse anti-PP2A catalytic subunit (1:1,000, Millipore), mouse anti-Hsp70 C7 (1:1,000, Abcam), mouse anti-lamin (1:200, Developmental Studies Hybridoma Bank), mouse anti-beta-tubulin, (1:200, Developmental Studies Hybridoma Bank), and mouse anti-H2B (1:1,000, Abcam). Secondary antibodies used were conjugated either with IRDye 680 or IRDye 800 (LICOR Bioscience) and incubated at a concentration of 1:10,000. Blots were visualized with Odyssey Infrared Imaging System (LICOR Biosciences).

### Immunoprecipitation

S2 cells expressing pMT-*lbr*-Flag-HA with or without pAc-*per*-V5-His were harvested 48 h after transfection. Protein extracts were prepared in EB2 (20 mM Hepes [pH 7.5], 100 mM KCl, 5% glycerol, 5 mM EDTA, 1 mM DTT, 0.1% Triton X-100, and 25 mM NaF) supplemented with complete EDTA-free protease and phosphatase inhibitor cocktail (MCE) and subsequently incubated with SureBeads to exclude nonspecific binding. Meanwhile, HA antibody (1:50, Cell Signaling Technology) was added to SureBeads and incubated at 4 °C for 4 h. Then, the extracts were incubated with HA antibody-bound beads at 4 °C overnight. Beads were magnetized to remove supernatant, washed 2 times in EB2, and resuspended in 1× loading buffer. Samples were analyzed by Western blotting as described above. Sodium dodecyl sulfate–polyacrylamide gel electrophoresis gel (6%) was used for analysis of PER protein. Primary antibodies used were rabbit anti-HA (1:1,000; Cell Signaling Technology), mouse anti-PP2A catalytic subunit (1:1,000, Millipore), mouse anti-lamin (1:200, Developmental Studies Hybridoma Bank), and guinea pig anti-PER (1:1,000, a gift from Dr. Joanna Chiu).

### Immunostaining

S2 cells were seeded on cell culture dishes with glass bottom and transfected with indicated plasmids. The cells were washed twice with 1×PBS, fixed with 3.7% paraformaldehyde for 10 min, and permeabilized with 0.5% Triton X-100 for 15 min. Thereafter, the cells were washed with 1×PBS and incubated with blocking buffer (1% bovine serum albumin and 0.1% Tween 20) for 30 min. Subsequently, the cells were incubated with primary antibodies at 4 °C overnight. After washing with 0.1% Tween 20 in PBS 4 times, the cells were incubated for 1 h with secondary antibody. Primary antibodies used are as follows: rabbit anti-PER (1:1,000, a gift from Dr. Joanna Chiu) and mouse anti-HA (1:1,000, Medical & Biological Laboratories). Secondary antibodies used were Alexa Fluor 488 goat anti-rabbit (1:1,000, Abcam) and Alexa Fluor 594 goat anti-mouse (1:1,000, Abcam). Nuclei were counterstained with Hoechst 33342 (Beyotime). Finally, the cells were rinsed with 0.1% Tween 20 in PBS 4 times and mounted with Vectashield Plus Antifade Mounting Medium (Vector Laboratories). The samples were scanned by the Olympus FV3000 confocal microscope with a 100× silicone oil objective.

### Live imaging

For fly brain imaging, flies were entrained in LD for 3 d and dissected on DD1 in chilled Schneider’s medium in less than 10 min under low-light conditions. A punched double-sided tape was used as a spacer on the slides to prevent flattening of the brains by coverslip. The brains were overlaid with a small amount of Vectashield Plus Antifade Mounting Medium and covered using a coverslip that was sealed with nail polish. The individual z-stack images of s-LNvs were acquired by the Olympus FV3000 confocal microscope with a 100× silicone oil objective.

For S2 cell imaging, S2 cells transfected with pAc-*per*-EGFP-V5-His and pMT-mCherry-Flag-His or pMT-*dbt-*mCherry-Flag-His were seeded on cell culture dishes with glass bottom. Hoechst 33342 was added into culture medium 10 min before imaging. Live S2 cells were imaged using the Olympus FV3000 confocal microscope 100× silicone oil objective with Z series and time series.

### 1,6-Hexanediol treatment

For treatment with 1,6-hexanediol (MACKLIN), Schneider’s *Drosophila* medium was prepared containing 10% 1,6-hexanediol. Target S2 cells were imaged with Z-stacks and then incubated in 10% 1,6-hexanediol medium for 1 min, followed by imaging again using the same conditions.

### FRAP

FRAP was performed on the Olympus FV3000 microscope with a 488-nm laser. PER foci were first identified using a 100× silicone oil objective. Acute light stimulation was achieved by utilizing the 488-nm laser line and stimulation module within the Olympus FV3000 imaging software. Regions of interest (ROIs) to be stimulated were drawn over fields of view prior to image acquisition. Reference ROIs of the same size were drawn adjacent to the cell. Following 1 baseline image, objects were bleached for 200 ms using 50% laser power (488-nm laser line), and images were collected every 2 s post-bleaching. The fluorescence signal measured in the ROIs were normalized to the change in total fluorescence as follows according to a previously published method: *I* = (*T*_0_/*T*_t_) * (*I*_t_/*I*_0_) [33]. *T*_0_ is the total intensity in the field before bleaching, while *T*_t_ is the total intensity in the field at timepoint *t*. *I*_0_ is the average intensity of ROIs before bleaching, while *I*_t_ the average intensity of ROIs at timepoint *t*.

### Protein purification

cDNAs encoding EGFP and PER-IDR EGFP were cloned into pET30a vector. The sequences were confirmed by sequencing. Plasmids of EGFP and PER-IDR EGFP were transformed into BL21 (DE3) and RosettaTM2 (DE3) *E. coli* cells, respectively. Isopropyl-β-D-thiogalactopyranoside was added at 0.5 mM concentration and incubated for 16 h at 15 °C or 4 h at 37 °C for EGFP and PER-IDR-EGFP, respectively. Pellets from 1L cells culture were collected and sonicated at 15 °C at 35% power for 2 s at intervals of 4.5 s for a total of 1 h for cells lysis. The lysates were centrifuged at 4 °C at 10,000 g for 30 min and the supernatants were used to purify target proteins by Ni nitrilotriacetic acid agarose (GE Healthcare).

### In vitro droplet assay

Recombinant protein was added to buffer (50 mM tris [pH 7.5] and 1 mM DTT) at varying protein or salt concentrations in the presence or absence of 10% PEG6000 (Sigma-Aldrich) and 10% 1,6-hexanediol. Glass slides were affixed with circular and single-sided tape with a hole drilled in the middle to stop limit liquid from spreading unchecked. The protein solutions were immediately loaded into the hole. The entire process (starting from sample preparation to the completion of imaging) does not exceed 5 min to prevent liquid evaporation and droplets from settling on the bottom of the glass.

### Confocal image analyses

Z-stack or time-lapse series were captured by the Olympus FV3000 confocal microscope. OIB files were then imported into Fiji as a composite image with a lossless 16-bit resolution per channel. A single Z-plane with the largest foci area or the brightest PER intensity was selected. Each focus was manually annotated for analysis. The mean pixel brightness (arbitrary unit) and geometric area (μm^2^) of the ROI were acquired using built in functions of Fiji software. For protein droplets assay, the area and number of droplets were analyzed by Fiji automatic counting.

### Statistical analyses

Student *t* test (GraphPad Prism) was used to analyze the differences between 2 groups for which data fits normal distribution. One-way analysis of variance (GraphPad Prism) was used to compare the differences between multiple groups that have only 1 explanatory variable. Two-way analysis of variance (ANOVA) (GraphPad Prism) was used to analyze the difference between 2 groups with 2 explanatory variables. Sample size used is consistent with previous literature utilizing similar assays, and samples were selected randomly. All data collected have been included and none was excluded. All replicates used are biological replicates.

## Data Availability

All data needed to evaluate the conclusions in the paper are present in the paper and/or the supplementary materials.
