# Supplementary material for: Phosphorylation Promotes the Accumulation of PERIOD Protein Foci
Source: Research (Wash D C). 2023 May 5;6:0139. doi: 10.34133/research.0139 (PMC10202380; doi:10.34133/research.0139)
Supplement: Supplementary 1 — Figs. S1 to S5 Table S1 [file research.0139.f1.docx]

**Supplementary Materials**


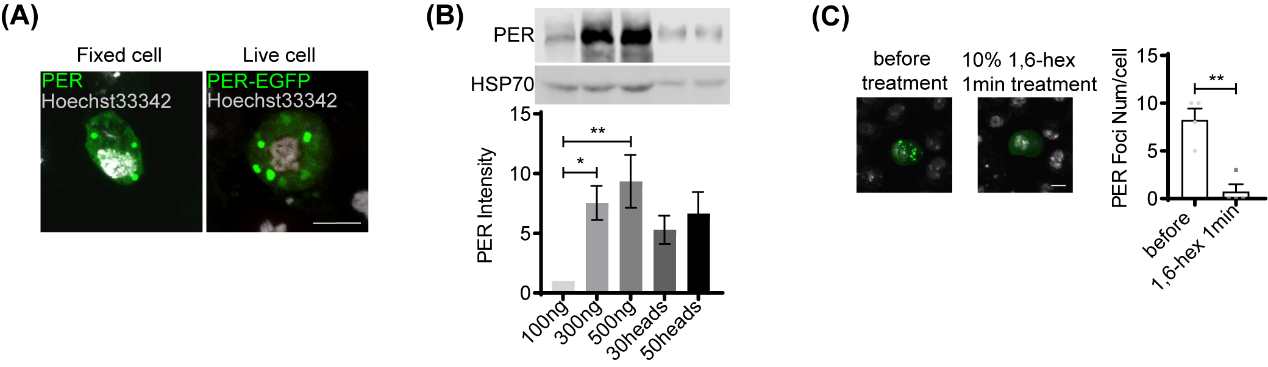


**Fig. S1. PER foci can be observed in S2 cells.**

(**A**) Left panel: representative confocal images of PER foci in S2 cells transfected with 100ng pAc-*per*-V5-HisB for 36hr and immunostained with hoechst33342 (gray) and PER antibody (green). Right panel: representative confocal images of PER foci in S2 cells transfected with pAc-*per*-EGFP-V5-HisB for 36hr and imaged live.

(**B**) Top panel: representative Western blots of protein extracts with equal concentration prepared from S2 cells transfected with pAc-*per*-V5-HisB for 36hr or fly heads of *w^1118^* flies collected on the first day of constant darkness (DD1). Bottom panel: quantification of PER level, which was normalized to that of HSP70. For each group, the value of the 100ng group was set to 1. n = 5. Error bars represent SEM. One-way ANOVA, Tukey’s multiple comparison test.

(**C**) Representative confocal images of PER foci in S2 cells transfected with 100ng pAc-*per*-EGFP-V5-HisB for 36hr and then treated with 10% 1,6-hexanediol for 1min. The plot shows PER foci number per cell. Error bars represent SEM. Student’s t-test. ***P* <0.01.

Scale bar, 5μm.


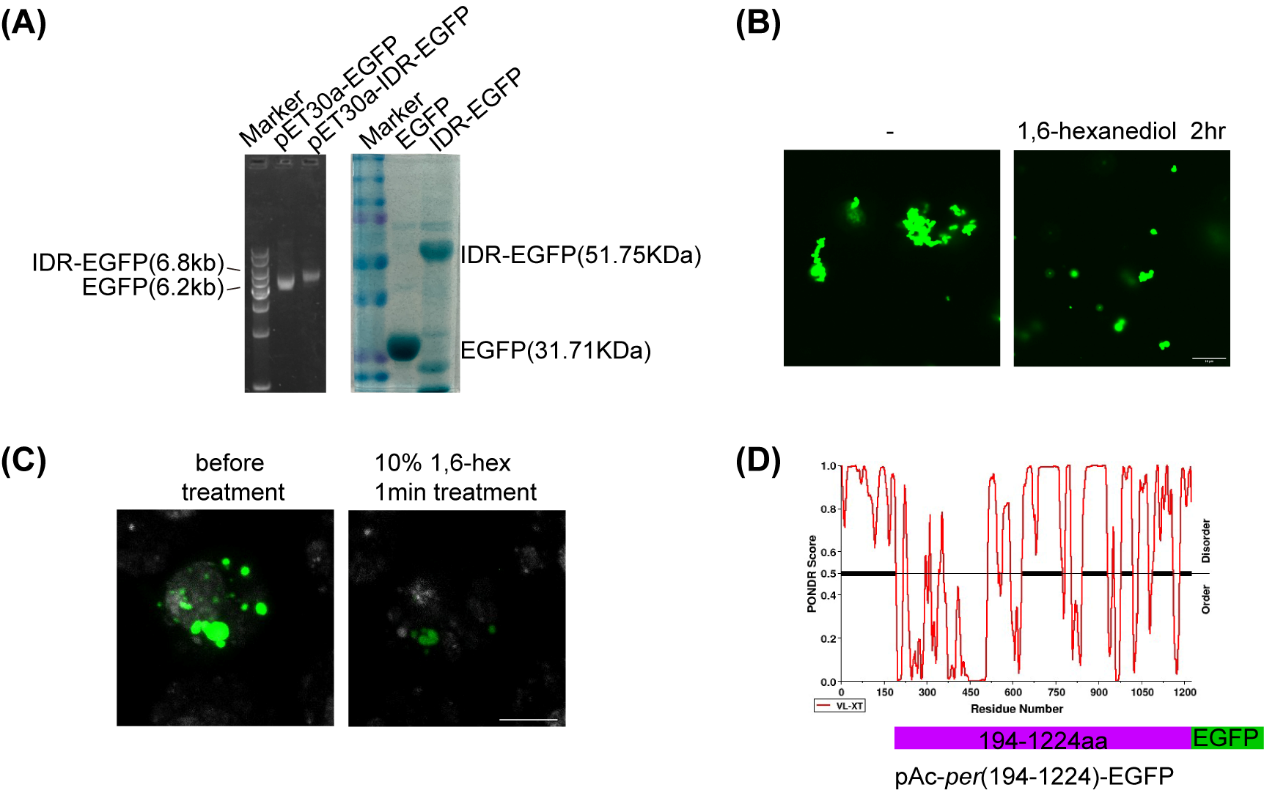


**Fig. S2. PER-IDR fused with EGFP can form phase-separated condensates in PEG.**

(**A**) Electrophoresis gel validation of plasmids encoding the recombinant protein (left) and the purified recombinant protein (right), respectively.

(**B**) Representative confocal images of 40μM PER-IDR-EGFP solution treated with 10% PEG6000 in the absence (left) or presence (right) of 10% 1,6-hexanediol treated for 2hr. Scale bar, 10μm.

(**C**) Top panel: disordered regions of PER protein indicated by black and bold lines. Bottom panel: schematics of recombinant plasmid.

(**D**) Representative confocal images of PER foci in S2 cells transfected with 100ng pAc-*per* (194-1224)-EGFP-V5-HisB for 36hr and then treated with 10% 1,6-hexanediol for 1min.

Scale bar, 5μm.


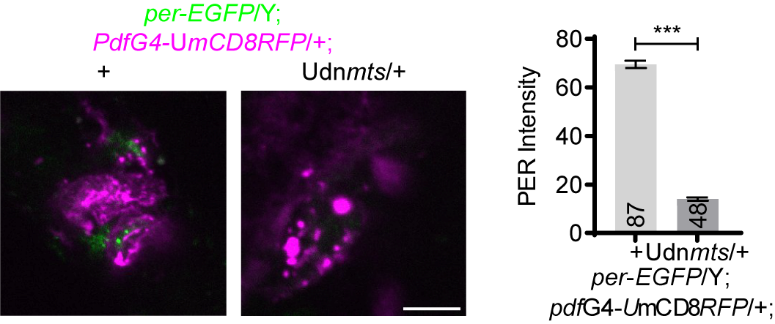


**Fig. S3. PER foci cannot be observed in dn*mts* expressing flies.**

Left: representative confocal images of PER foci in the s-LNvs of *per-*EGFP/Y;*Pdf*GAL4*-*UASmCD8RFP*/+* flies over expressing dn*mts* and control flies at CT0 on DD1. Green, PER-EGFP; magenta, mCD8RFP. Scale bar, 5μm. G, GAL. U, UAS. Right: quantification of PER protein intensity in the s-LNvs of *per-*EGFP/Y;*Pdf*GAL4*-*UASmCD8RFP*/+* flies over expressing dn*mts* and control flies. n refers to the number hemispheres and is indicated on the bars. Error bars represent SEM. Student’s t-test. **P* <0.01, ***P* <0.01, ****P* <0.001.


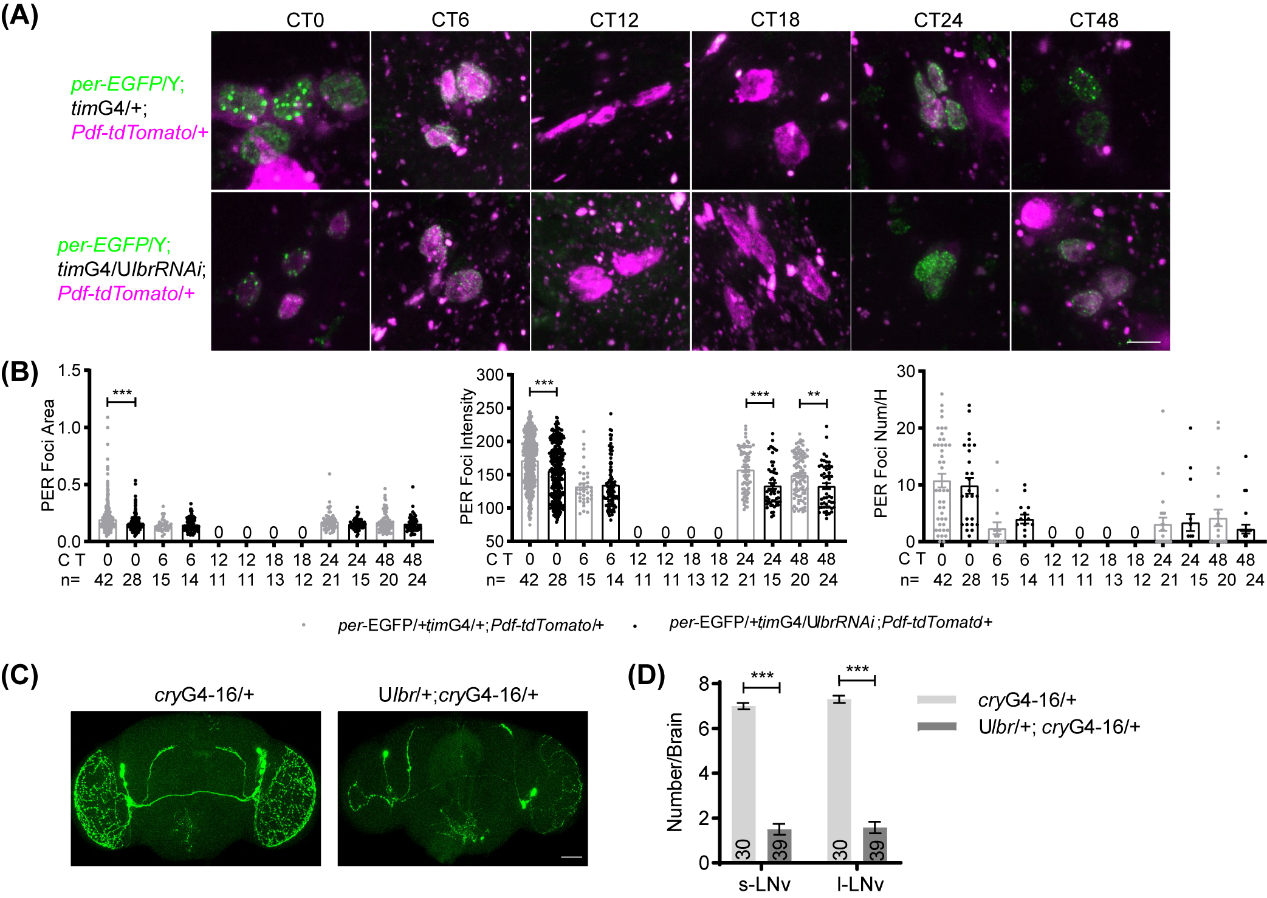


**Fig. S4. Knocking down *lbr* reduces PER foci size and intensity.**

(**A**) Representative confocal images of PER foci in the s-LNvs of *per*-EGFP/Y;*tim*G4/U*lbrRNAi*;*Pdf-tdTomato*/+ and control flies at indicated time points. Green, PER-EGFP; magenta, tdTOMATO. Scale bar, 5μm.

(**B**) Quantification of PER foci area, intensity and number per hemisphere in (A). n refers to the number of hemispheres.

(**C**) Representative confocal images of PDF immunostaining which labels s-LNvs and large ventral lateral neurons (l-LNvs) in U*lbr*/+;*cry*G4-16 and control flies. Scale bar, 50μm.

(**D**) Quantification of the number of s-LNvs and l-LNvs per brain in (C). n refers to the number of brains and is indicated on the bars.

Error bars represent SEM. Student’s t-test. **P* <0.01, ***P* <0.01, ****P* <0.001. G, GAL4. U, UAS.


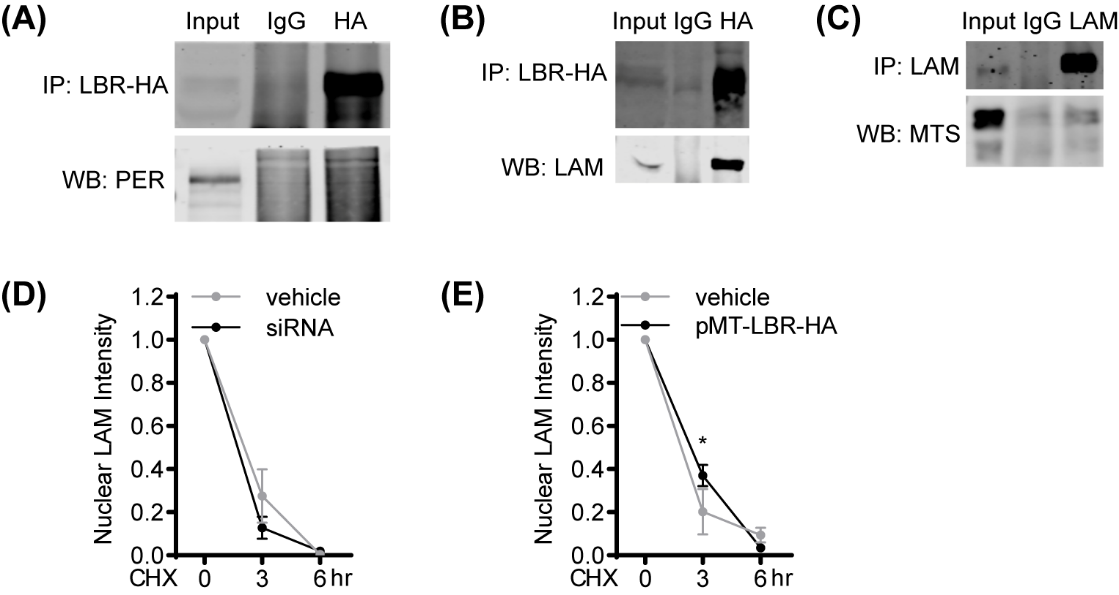


**Fig. S5. LBR binds to and stabilizes lamin.**

(**A**) Representative Western blots (WB) of protein extracts and immunoprecipitates prepared from S2 cells co-transfected with pMT-*lbr*-Flag-HA and pAc-*per*-V5-HisB. LBR was immunoprecipitated (IP) with HA antibody, and rabbit IgG was used as control.

(**B**) Representative Western blots (WB) of protein extracts and immunoprecipitates prepared from S2 cells transfected with pMT-*lbr*-Flag-HA. LBR was immunoprecipitated with HA antibody, and rabbit IgG was used as control.

(**C**) Representative Western blots (WB) of protein extracts and immunoprecipitates prepared from S2 cells. LAM was immunoprecipitated with LAM antibody, and mouse IgG was used as control. MTS was detected by Western blotting using MTS antibody.

(**D**) Quantification of LAM level in knocking down *lbr* and control groups in (Fig. 4 E).

(**E**) Quantification of LAM level in over-expressing *lbr* and control groups in (Fig. 4 H).

n=3. Error bars represent SEM. Two-way ANOVA, Sidak’s multiple comparison test was used for (D and E). **P* <0.05, ***P* <0.01, ****P* <0.001. G, GAL. U, UAS. LAM, lamin.

Table S1. LBR participates in regulating locomotor rhythm.

| Genetype | N | Rhythmicity% | Periods(hr) | Power |
| --- | --- | --- | --- | --- |
| U*lbrRNAi*/+ | 88 | 98.86 | 23.51±0.02 | 106.07±5.04 |
| *Pdf*G4-U*dcr2*/+ | 74 | 100.00 | 24.47±0.05 | 168.72±3.78 |
| *Pdf*G4-U*dcr2*/U*lbrRNAi* | 74 | 100.00 | 24.25±0.05^#^ | 138.04±5.19^**###^ |
| U*dcr2*/+; *cry*G4-16/+ | 81 | 100.00 | 25.73±0.11 | 96.71±6.27 |
| U*dcr2*/U*lbrRNAi*; *cry*G4-16/+ | 108 | 92.59 | 26.09±0.10^###^ | 49.27±4.24^***###^ |
| *tim*G4/+; U*dcr2*/+ | 101 | 96.04 | 24.33±0.07 | 61.04±4.44 |
| *tim*G4/*UlbrRNAi*; U*dcr2*/+ | 129 | 74.42 | 26.28±0.70^***###^ | 16.88±2.14^***###^ |
| U*lbr*/+ | 90 | 98.89 | 23.44±0.06 | 146.21±6.45 |
| *Pdf*G4/+ | 83 | 100.00 | 24.32±0.04 | 163.28±4.86 |
| *Pdf*G4/U*lbr* | 103 | 94.17 | 24.60±0.10^***###^ | 75.55±4.28^***###^ |
| *cry*G4-16/+ | 84 | 100.00 | 24.65±0.06 | 105.98±5.18 |
| U*lbr/+*; *cry*G4-16/+ | 115 | 60.87 | 27.87±1.00^***###^ | 11.24±1.43^***###^ |
| *tim*G4/+ | 67 | 100.00 | 24.66±0.10 | 105.88±5.18 |
| *tim*G4/U*lbr* | lethal |  |  |  |

*One-way ANOVA compared to UAS*RNAi* control lines, **P* <0.05, ***P* <0.01, ****P* <0.001.

^#^One-way ANOVA compared to control lines with GAL4 and UAS*dcr2*, ^#^*P* <0.05, ^##^*P* <0.01, ^###^*P* <0.001.

*Dicer2* (*dcr2*) is co-expressed to enhance the effects of RNAi. G, GAL4; U, UAS.

**Video 1. FRAP assay of PER foci in PER-EGFP expressing S2 cells.**

**Video 2. Fusion of PER-IDR droplets *in vitro*.**
